# An AFM Approach Applied in a Study of α-Crystallin Membrane Association: New Insights into Lens Hardening and Presbyopia Development

**DOI:** 10.3390/membranes12050522

**Published:** 2022-05-14

**Authors:** Nawal K. Khadka, Raju Timsina, Laxman Mainali

**Affiliations:** 1 Department of Physics, Boise State University, Boise, ID 83725, USA; nawalkhadka@boisestate.edu (N.K.K.); rajutimsina@boisestate.edu (R.T.); 2 Biomolecular Sciences Graduate Program, Boise State University, Boise, ID 83725, USA

**Keywords:** atomic force microscopy, α-crystallin membrane interaction, mechanical properties, breakthrough force, topographical image, membrane elasticity, lens hardening

## Abstract

The lens of the eye loses elasticity with age, while α-crystallin association with the lens membrane increases with age. It is unclear whether there is any correlation between α-crystallin association with the lens membrane and loss in lens elasticity. This research investigated α-crystallin membrane association using atomic force microscopy (AFM) for the first time to study topographical images and mechanical properties (breakthrough force and membrane area compressibility modulus (K_A_), as measures of elasticity) of the membrane. α-Crystallin extracted from the bovine lens cortex was incubated with a supported lipid membrane (SLM) prepared on a flat mica surface. The AFM images showed the time-dependent interaction of α-crystallin with the SLM. Force spectroscopy revealed the presence of breakthrough events in the force curves obtained in the membrane regions where no α-crystallin was associated, which suggests that the membrane’s elasticity was maintained. The force curves in the α-crystallin submerged region and the close vicinity of the α-crystallin associated region in the membrane showed no breakthrough event within the defined peak force threshold, indicating loss of membrane elasticity. Our results showed that the association of α-crystallin with the membrane deteriorates membrane elasticity, providing new insights into understanding the molecular basis of lens hardening and presbyopia.

## 1. Introduction

Human lenses are the avascular tissues that provide the geometrical and physiological arrangement for focusing light on the retina. Lens fiber cells containing a very high concentration of protein, mostly crystallin [1], are meticulously arranged in the lens to provide a lifelong mechanism for light refraction. As eye lens cells are generated continuously throughout life, older cells become elongated and constricted at the center, forming a nucleus, and the newly grown cortical fiber cells form a layer over the nucleus [2]. Presbyopia—the loss of the lens’ accommodative ability to focus on nearby objects—begins in most humans around and beyond the age of 40, regardless of any prior vision condition. Several factors are proposed to explain the underlying cause of presbyopia, including lens hardening [3,4,5,6,7], lens growth [8], aging of the ciliary muscle [9], lens capsule [10], and vitreous [11]. Recent discoveries suggest that age-related loss in lens elasticity is the preeminent factor for presbyopia [3,4,6,12,13,14]. Both ex vivo [15] and in vivo [16] experiments on the mechanical properties of the human lens have indicated that the tissues in the central nuclear region are stiffer than the tissues in the peripheral region of the lens [3,17]. Similarly, the nuclear cells are much stiffer than the cells in the cortical region [18]. The dramatic loss of lens elasticity (i.e., increased lens stiffness) occurs most prominently in the fourth to fifth decade of life [3,4,5,19,20]. Cataractous lenses follow a similar trend, with older cataractous lenses being stiffer than younger ones [21]. However, cataractous lenses are substantially stiffer than clear lenses in the same age group [21]. As the lens loses elasticity with age, α-crystallin concentration decreases in the lens cytoplasm, where α-crystallin associates with other crystallins, forming a higher molecular weight complex (HMWC) [4,5,22,23,24,25,26,27]. The HMWC further associates with the fiber cell plasma membrane [25,26,28,29]. It has also been reported that mild thermal stress in the young human lens causes a large-scale association of α-crystallin with the lens membrane [30]. It is likely that the association of α-crystallin and HMWC with the fiber cell plasma membrane reduces the ability of individual fiber cells to change shape and that this is accompanied by lens hardening and presbyopia development. Some researchers have also pointed out that presbyopia might be an early symptom of nuclear cataracts [31].

Several studies have investigated the interaction of α-crystallin with the lens membranes [32,33,34,35,36,37,38] and lipid vesicles [32,34,39,40,41,42,43,44]. Intrinsic phospholipids in membranes serve as primary association sites for α-crystallin [32,33,41]. Multiple studies have suggested that α-crystallin associates with the membrane via hydrophobic interaction [45,46,47,48], modulating the physical properties of the membrane [26,32,39,40,48,49,50]. The membrane insertion ability of αA-crystallin correlates with its oligomeric size, suggesting that oligomeric size may be the structural basis for the localized association of αA-crystallin with the membrane [47]. Our electron paramagnetic resonance (EPR) studies demonstrated that the association of α-crystallin with lipids and cholesterol-containing lipid membranes changes the physical properties (mobility parameter, maximum splitting, and hydrophobicity) of membranes [39,40,48,49,50], that lipid composition strongly modulates the association of α-crystallin with membranes [26,40,48], and that an increase in cholesterol (Chol) content, with the formation of cholesterol bilayer domains (CBDs), within the Chol/lipid membranes inhibits the association of α-crystallin with the membranes [48,49]. Atomic force microscopy (AFM) was used previously to measure the stiffness of the lens cell [18], capsule [51], and whole lens [52]. AFM is a versatile instrument for analyzing various aspects of proteins, providing new understandings of molecular mechanisms and making significant contributions to protein biology [53]. A recent publication [53] reviewed the versatility of AFM from a variety of viewpoints, including single particle force spectroscopy, morphological imaging, mechanical unfolding processes, and high-speed imaging of single proteins. Recently, we developed the AFM approach and measured the elasticity of the high cholesterol-containing membrane relevant to the eye lens membrane [54]. However, the interaction of α-crystallin with the supported lipid membrane (SLM) using the AFM approach has not been investigated. In this study, we used the AFM approach for the first time to study the topographical image and mechanical properties (breakthrough (BT) force and elasticity) of the membrane after α-crystallin association and provide new insights through an elucidation of the molecular basis of lens hardening and presbyopia development.

## 2. Materials and Methods

### 2.1. Materials

Bovine eye lenses were purchased from Pel-Freez Biologicals (Rogers, AR, USA) and stored at −80 °C immediately upon receipt, and 1-palmitoyl-2-oleoyl-sn-glycero-3-phosphatidylcholine (POPC) lipid dissolved in chloroform was purchased from Avanti polar lipids (Birmingham, AL, USA). POPC was chosen as a model lipid because phosphatidylcholine is dominant in the lens membrane of short-life span animals [55]. Analytical grade NaN_3_, CaCl_2_, MgCl_2_, HEPES, and NaCl were purchased from Sigma-Aldrich (St. Louis, MO, USA), and Tris base was purchased from Fisher bioreagents. The elution buffer contained 1 mM NaN_3_, 20 mM Tris-HCl, and 150 mM NaCl and had a pH of 7.9. Buffer A contained 10 mM HEPES and 150 mM NaCl, with a pH 7.4, while buffer B contained 10 mM HEPES and 150 mM NaCl, pH 7.4, with 5 mM CaCl_2_ or 10 mM MgCl_2_. All SLMs were prepared on buffer B containing 5 mM CaCl_2_ unless specified otherwise. Approximately 5 mL of divalent salt-free buffer (buffer A) was used for flushing out unfused vesicles before imaging, as described previously [54].

### 2.2. α-Crystallin Extraction and Purification

A single bovine lens was separated into the cortical and nuclear regions based on tissue consistency after decapsulation. Soluble proteins were extracted from the cortex using a previously described protocol [56]. Briefly, after homogenizing cortical tissues in the elution buffer, cell debris was pelleted by centrifuging at 18,000× *g* for 15 min at 4 °C (Beckman Coulter, Brea, CA, USA). The supernatant containing all soluble proteins was filtered using a syringe filter containing 0.22 µm pores. Then, 5 mL of filtered supernatant was loaded in a Hiload 16/600 Superose 6 pg gel filtration column connected to an AKTA go protein purification system for size exclusion chromatography. The solution was eluted at a 1 mL/min flow rate, and protein fractionations were monitored at 280 nm absorbance. α-Crystallin collected in the rotatory fraction collector was concentrated using Amicon Ultra-15 filters by centrifuging at 3024× *g* at 4 °C, dialyzed in buffer A, and stored at 4 °C until further use. The purity of isolated α-crystallin was confirmed by sodium dodecyl sulfate–polyacrylamide gel electrophoresis (SDS-PAGE). The concentration of α-crystallin was estimated using an average extinction coefficient (ε) of 14,325 M^−1^ cm^−1^ and an average molecular weight of the single subunit of 19.85 KDa, where average extinction coefficient and molecular weight were estimated using αA:αB = 3:1 (for adult bovine lens [57]) from the values (αA = 19.79 kDa, αB = 20.04 kDa, ε_A_ = 14,440 M^−1^ cm^−1^, ε_B_ = 13,980 M^−1^ cm^−1^) obtained using the ProtParam tool [58].

### 2.3. AFM Experiment

SLM made of POPC was prepared using a procedure described in our recent paper [54]. After the initial imaging and force curve acquisition on the SLM, ~300 µL of 0.1 mg/mL α-crystallin solution in buffer A was passed through the inlet of the fluid cell (fluid cell well volume was ~75 µL) in SLM and incubated for ~30 min. Images and force curves were taken at different time intervals to study the interaction of α-crystallin with the membrane. Around 10 µL of 0.025 mg/mL α-crystallin was deposited on a freshly cleaved mica disk, dried in the open air, subsequently washed with ~500 µL water ten times, re-dried, and scanned using AFM to observe the distribution and structure of α-crystallin in the absence of membrane. All experiments were performed at room temperature.

We used a Bruker Multimode VIII (Santa Barbara, CA, USA) AFM equipped with an E-scanner and controlled by a nanoscope V controller to image and capture force curves. Commercially available DNP-S probes with an estimated tip-end radius of 9 nm to 22 nm (nominal radius 10 nm) and a spring constant of 0.4 N/m to 0.6 N/m (nominal 0.35 N/m) were used in a fluid cell or standard air probe holder. The tip-end radiuses of all probes used in the experiment were estimated by analyzing height images of a titanium characterizer (Model PFQNM-SMPKIT-12M) with many sharp grain features, which were imaged before the experiments were performed and analyzed using the tip-quantification function provided in Nanoscope analysis 1.9 (Bruker, Santa Barbara, CA, USA) software. After calibrating the deflection sensitivity, the spring constant was calibrated using the embedded thermal noise tool before each experiment. Five cantilevers in total were used in our experimental process, each of which was tuned before each experiment. The images were acquired with 384 × 384 samples per line at a 1 Hz scan rate and 2560 data points in each force curve. At least 400 force curves, with adjacent curves laterally offset by at least 100 nm, were acquired in each SLM image. Images were flattened in first or second order in Nanoscope analysis 1.9 software and further processed with a homemade script using Matlab R2018b, MathWorks (Natick, MA, USA).

## 3. Results

### 3.1. Topographical Images of Time-Dependent α-Crystallin Membrane Interaction

**Figure 1:**
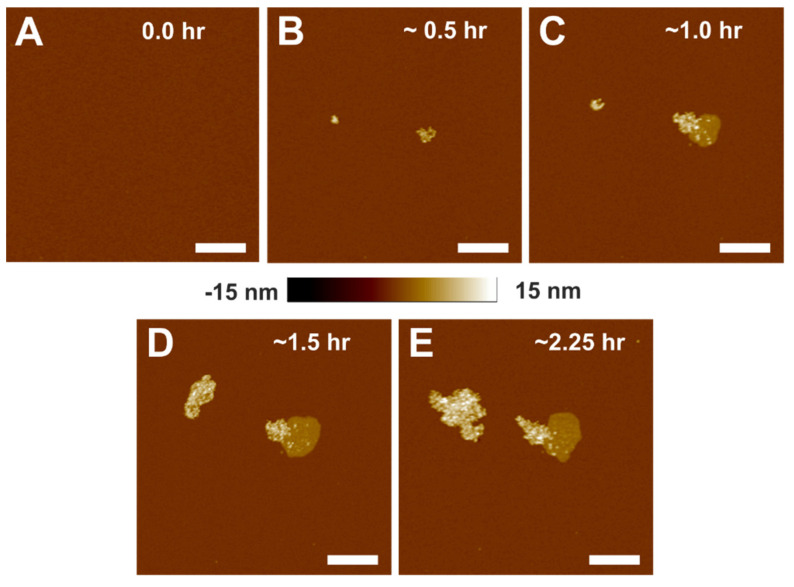
Topographical images of the interaction of α-crystallin with SLM with increasing incubation time. (**A**) The membrane before adding α-crystallin. (**B**–**E**) The membrane after adding α-crystallin, showing how the size of α-crystallin aggregates increases with time. As shown in (**C**–**E**), the intermediate complex formed, as represented by the intermediate height color, suggests a strong interaction of α-crystallin with the membrane. Image scale: 1 µm.

We investigated the time-dependent association of α-crystallin with the SLM by capturing topographical images using AFM. After confirming the defect-free complete SLM, as shown in Figure 1A, α-crystallin in buffer A was deposited on the SLM to investigate the time-dependent association, as shown in Figure 1B–E. Surprisingly, α-crystallin oligomers did not uniformly associate with the membrane; instead, they clustered together as an aggregate (Figure 1B). With a longer incubation time, surprisingly, α-crystallin oligomers associated deeper in the membrane, forming α-crystallin–membrane complexes, as visualized by the intermediate color (light brown) shown in Figure 1C. The size of such complexes increased with increased incubation time, as shown in Figure 1C–E. The time-dependent association of α-crystallin with POPC small unilamellar vesicles was observed in our previous study using the EPR method [39]. Cobb and Petrash et al. [45] also reported the time-dependent association of αA- and αB-crystallins, as well as an αA:αB = 3:1 heteromeric complex, with native bovine lens membranes using the fluorescence method. We believe that the α-crystallin–membrane complex is formed by the strong interaction of α-crystallin with the phospholipid membrane, which is observed only around the pre-existing aggregate of α-crystallin oligomers and not on its own. This being the case, a possible mode of interaction of α-crystallin with the membrane is hydrophobic interaction between the hydrophobic core of the membrane and hydrophobic residues of α-crystallin oligomers, implying that some part of the α-crystallin oligomer is inserted into the membrane, as suggested by Bloemendal et al. [59]., To the best of our knowledge, our study is the first to visualize α-crystallin membrane interaction using AFM. How much of the α-crystallin oligomer inserts into the membrane is unclear and needs further investigation. Numerous studies [45,46,47,48] have reported the hydrophobic interaction of α-crystallin with membranes. Previously, we suggested that the hydrophobic regions exposed on the outer surface of α-crystallin oligomers are inserted into the lipid and Chol/lipid membranes [27,41,50]. It has also been reported that denatured α-crystallin associates deep into the membrane [29,46,60].

### 3.2. Mechanical Properties of Membranes with α-Crystallin Association

**Figure 2:**
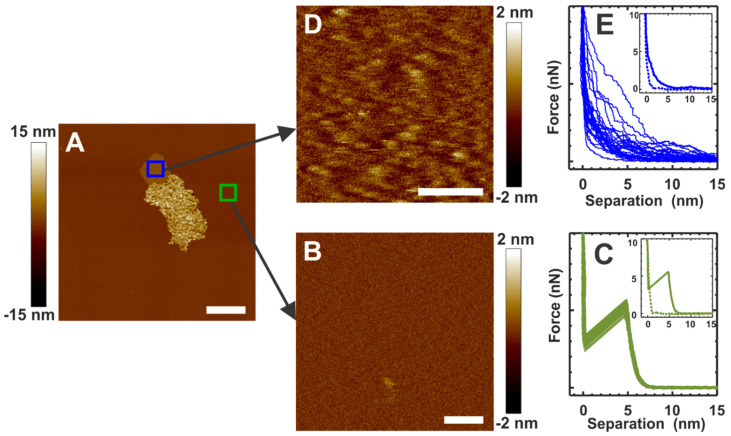
(**A**) α-Crystallin interacting with the SLM at around 1 h incubation time. (**B**) AFM image of the α-crystallin-free membrane region, identified by the green square in (**A**). (**C**) Representative force curves in the membrane region, as shown in (**B**). (**D**) The AFM image collected in the blue square region in (**A**) shows a closer view of the structural details of the α-crystallin membrane region with strong α-crystallin membrane interaction and the submersion of α-crystallin into the membrane. Bumps in (**D**) suggest α-crystallin oligomers submerged in the membrane. (**E**) Representative force curves for the membrane region of (**D**). Representative complete force curves are shown in (**C**,**E**) insets, with solid and dotted curves representing approach and retract curves, respectively. Image scales: 1 µm for (**A**); 0.1 µm for (**B**,**D**).

We collected force curves for the α-crystallin membrane interaction-free region and the α-crystallin submerged region in the membrane, as shown by the green and blue squares, respectively, in Figure 2A, to investigate the modulation of the mechanical properties of the membrane after α-crystallin association. Figure 2B shows the topographical image of a closer view of the membrane, indicated by the green square in Figure 2A. Figure 2C shows the corresponding force curves collected in the membrane region shown in Figure 2B. The force curves shown in Figure 2C are the typical force curves of the membrane, displaying rupture events with an average BT force of 5.20 ± 0.21 nN, as observed previously [54]. The clear rupture events imply that the elastic nature of the membrane is maintained. The inset in Figure 2C displays a representative complete force curve collected in the membrane region shown in Figure 2B, with the solid green and dotted green curves representing the approach and the retract sections of the complete force curves, respectively. Figure 2D shows the topographical image of a closer view of the α-crystallin submerged membrane region indicated by the blue square in Figure 2A. We speculate that the slightly raised bumps in the image shown in Figure 2D correspond to the α-crystallin oligomers. Figure 2E shows the corresponding force curves collected in the α-crystallin submerged region in the membrane shown in Figure 2D. The inset in Figure 2E displays representative complete force curves collected in the membrane region shown in Figure 2D, with the solid blue and dotted blue curves representing the approach and the retract sections of the complete force curves, respectively. The force curves obtained in the α-crystallin submerged membrane region did not display rupture events or typical membrane force curves, as shown in Figure 2E, suggesting a loss of elastic properties in the membrane. Unless otherwise stated, all the force curves displayed in this manuscript are the approach sections of complete force curves.

**Figure 3:**
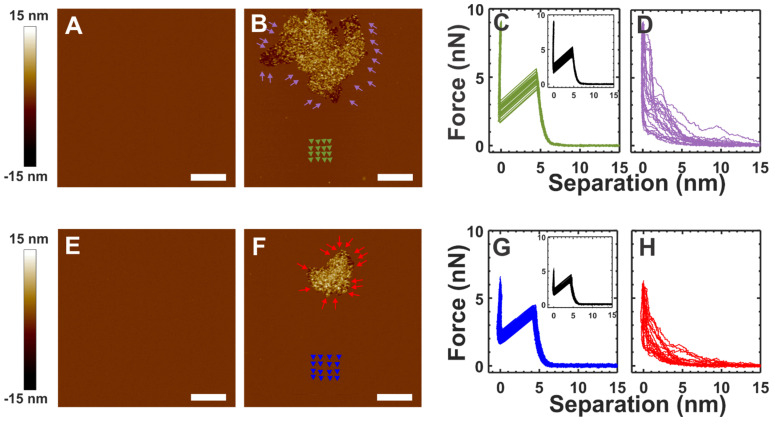
α-Crystallin interacting with the SLMs prepared with different buffers. The top and bottom rows correspond to images and force curves of the membrane prepared using buffer B with 5 mM CaCl_2_ and 10 mM MgCl_2_, respectively, though ~5 mL of divalent salt-free buffer (buffer A) was used for flushing out unfused vesicles before imaging, as described previously [54]. (**A**,**E**) The SLM without α-crystallin. (**B**,**F**) α-Crystallin in buffer A incubated with SLM for ~40 min. (**C**) Force curves (green curves) were obtained for the membrane region around the green arrowheads in (**B**), while the inset force curves (black curves) were obtained for the control membrane shown in (**A**). (**D**) Force curves (purple curves) were obtained around the α-crystallin associated membrane region, as shown by the purple arrows in (**B**). (**G**) Force curves (blue curves) were obtained for the membrane region around the blue arrowheads in (**F**), while the inset force curves (black curves) were obtained in the control membrane shown in (**E**). (**H**) Force curves (red curves) were obtained around the α-crystallin associated membrane region, as shown by the red arrows in (**F**). The arrows/arrowheads in the membrane show the approximate representative positions of the force curves taken. The complete force curves of (**C**,**G**) are similar to those in the Figure 2C inset, while the complete force curves of (**D**,**H**) are similar to those in the Figure 2E inset. Image scale: 1 µm.

The top and bottom rows in Figure 3 show the results based on the SLM prepared with buffer B containing 5 mM CaCl_2_ and 10 mM MgCl_2_, respectively. Figure 3B,F show the topographical images obtained after the incubation of α-crystallin with the SLM shown in Figure 3A,E, respectively, for about 40 min. The colored arrow/arrowheads in Figure 3B,F show the approximate representative positions for the corresponding colored force curves shown in Figure 3C,D,G,H, whereas the insets (black) in Figure 3C,G correspond to the force curves obtained from the membranes (without α-crystallin) shown in Figure 3A,E, respectively. The average BT forces for the green curves and black inset curves in Figure 3C were 5.20 ± 0.21 nN and 5.30 ± 0.26 nN, respectively. The green curves shown in Figure 3C were obtained from the membrane region away from the α-crystallin associated region indicated by green arrowheads in Figure 3B. Atypical force curves with no rupture events, as seen in Figure 3D, were obtained near the vicinity of the α-crystallin associated membrane region, as indicated by purple arrows in Figure 3B, suggesting a loss of membrane elasticity. Similar properties were obtained in the SLM prepared with MgCl_2_ in buffer B (Figure 3G,H). The representative force curves in the control membrane (Figure 3E) and the membrane indicated by the blue arrowhead in Figure 3F are shown in Figure 3G as the black curves (inset) and blue curves, possessing an average BT force of 3.98 ± 0.24 nN and 3.84 ± 0.29 nN, respectively. The force curves in the vicinity of the α-crystallin associated membrane region, represented by red arrows in Figure 3F, displayed atypical membrane force curves with no actual rupture events, as shown in Figure 3H, suggesting loss of membrane elasticity. Although a slightly lower BT force was detected for the SLM prepared with Mg^2+^ ions compared to that prepared with Ca^2+^ ions (Figure 3C,G), the effects of α-crystallin membrane association on membrane mechanical properties were identical.

**Figure 4:**
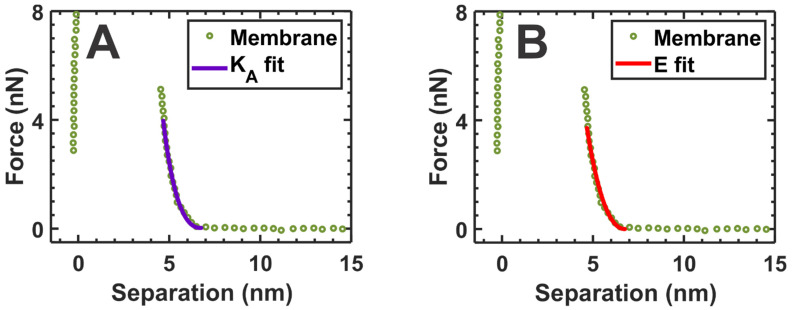
(**A**) Representative fit of Equation (1) with the force curve to calculate the membrane’s area compressibility modulus (*K_A_*), where *K_A_* is a measure of membrane elasticity [54]. (**B**) Representative fit of Equation (2) with the force curve to calculate the Young’s modulus (*E*), where E is a measure of membrane elasticity [64]. The representative force curve is taken from the membrane region indicated by the green arrowheads in Figure 3B.

We calculated the area compressibility modulus (*K_A_*) of the membrane to obtain further information about the mechanical properties of the SLM. Assuming a spherical tip-end and a free-standing SLM, the tip force in the elastic regime of the force curve can be fitted quadratically with the tip–mica separation distance given by the following equation [54,61]:

(1) $$F=\pi K_{A}R{\left(\frac{D-s}{s}\right)}^{2}$$

where *D* is the thickness of the SLM, including the water layer residing between mica and the SLM [54,62], *s* is the tip–mica separation distance, and *R* is the tip-end radius of the AFM tip. Thus, using Equation (1), we fitted the elastic regime of the approach curves by taking the approach section of the force curves after the AFM tip touched the membrane surface until it first reached 80% of the BT force of the membrane and estimated the *K_A_*, which is the measure of membrane elasticity [54]. Since the validity of Equation (1) is only within the elastic limit, we fitted 80% of the elastic region in the force curve, starting from the point of contact. A representative fit of this Equation (1) is shown in Figure 4A, where a representative force curve is taken from the membrane region indicated by green arrowheads in Figure 3B. By fitting the representative force curves shown in the Figure 3C inset (black curves), the average *K_A_* for the membrane before adding α-crystallin was estimated to be 268.05 ± 66.1 mN/m. A similar average value for the *K_A_*, 274.89 ± 58.4 mN/m, was obtained by fitting the force curves shown in Figure 2C for the SLM region where α-crystallin was not associated. Furthermore, the force curves in the membrane obtained with AFM can be described by a modified Hertz model [62,63]:

(2) $$F=\frac{16}{9}ER^{1/2}\delta^{3/2}\left(1+0.884\rho +0.781\rho^{2}+0.386\rho^{3}+0.0048\rho^{4}\right)$$

where *E* is the Young’s modulus—a measure of membrane elasticity; *δ* is indentation depth, defined by *D* − *s*; and ρ is a dimensionless parameter defined by $\sqrt{R\delta }/h$. Here, *h* is the membrane thickness and is defined as *h* = *D* − t_w,_ with D being the distance between the mica to the initial point of contact by the AFM tip and t_w_ being the water layer thickness, which is assumed to be 2 nm [64]. *R* is the tip-end radius of the AFM tip, and *s* is the tip–mica separation distance. To ensure the fit only in the elastic regime, we fitted a region in the force curve (i.e., an approach curve until 80% of BT force) similar to that used for the *K_A_* calculation above. An example of the fit for Equation (2) is shown in Figure 4B.

We also estimated the *K_A_* and *E* values for the membrane before and after adding α-crystallin. The estimated values of *K_A_* and *E* for the membrane before adding α-crystallin, as shown in Figure 3A, were 268.05 ± 66.1 mN/m and 27.9 ± 5.8 MPa, respectively. The *K_A_* and *E* values after the addition of α-crystallin in the membrane region far away from the vicinity, as indicated by the green arrowhead in Figure 3B, were estimated to be 291.2 ± 69.2 mN/m and 29.4 ± 6.2 MPa, respectively. Similar *K_A_* and *E* values were obtained for the membrane shown in Figure 3A and the membrane region indicated by green arrowheads in Figure 3B. Similarly, *K_A_* and *E* values before the addition of α-crystallin in the membrane, as shown in Figure 3E, were estimated as 271.7 ± 77 mN/m and 27.3 ± 7.4 MPa, respectively. In addition, *K_A_* and *E* values after the addition of α-crystallin in the membrane, as shown in Figure 3F (the membrane region indicated by the blue arrowheads), were estimated to be 281.2 ± 87.9 mN/m and 31.4 ± 8.3 MPa, respectively. Similar *K_A_* and *E* values were obtained for the membrane shown in Figure 3E and the membrane region indicated by blue arrowheads in Figure 3F. The estimated *K_A_* and *E* values for the membrane agree well with the values reported in the literature [54,62,64]. However, the force curves shown in Figure 2E that were obtained for the α-crystallin submerged membrane region shown in Figure 2D did not exhibit typical elastic natures and puncture events (within the set force threshold limit), suggesting a loss of membrane elastic behavior. Similarly, the force curves shown in Figure 3D,H obtained in the vicinity of the α-crystallin associated membrane regions indicated by purple arrows in Figure 3B and red arrows in Figure 3F, respectively, did not exhibit typical elastic natures and puncture events (within the set force threshold limit), suggesting a loss of membrane elastic behavior. Thus, for the regions indicated by the purple and red arrows in Figure 3B,F, respectively, and the region shown in Figure 2D, *K_A_* and *E* could not be estimated based on the obtained force curves.

### 3.3. Distribution of α-Crystallin Oligomers

**Figure 5:**
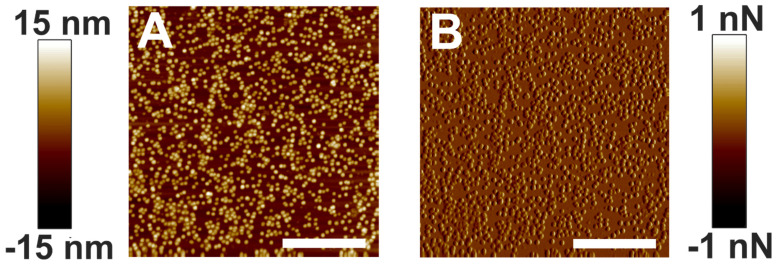
α-Crystallin adsorbed in mica. (**A**) Height image of the α-crystallin oligomers adsorbed in the mica disk. (**B**) Corresponding peak force error image. Image scale: 1 µm.

Figure 5A,B are the height and peak force error images of electrostatically associated α-crystallin on a mica surface acquired with AFM. As expected, polydisperse α-crystallin oligomers and a close association of such oligomers are seen. Interestingly, a comparable image of negatively stained bovine lens α-crystallin was reported previously using an electron microscope [65]. The present paper reports AFM imaging of α-crystallin oligomers (Figure 5) for the first time. Bovine lens α-crystallin exists as oligomers of 300–900 kDa [37,57] with large hydrophobic regions, allowing for the possibility of self-interaction [66]. Although we did not use any staining, the size of α-crystallin oligomers as determined by AFM might have been affected by the tip radius, setpoint force, pixel size, and ambient conditions. Since the purpose of our study was to determine how α-crystallin association with the membrane modulated the mechanical properties of the membrane, we did not report the actual size of the α-crystallin oligomers. However, we displayed the overall distribution of α-crystallins used in our experiment. The distributions of α-crystallin oligomers interacting with the membrane, as shown in Figure 1, Figure 2 and Figure 3, and on the mica surface, as shown in Figure 5, were significantly different. 

## 4. Discussion

Interaction of α-crystallin with the plasma membrane in the lens of the human eye around the fourth to fifth decade of life has been believed to progress cataract formation and increase lens stiffness [3] which is the likely cause of the failure of proper accommodation known as presbyopia. Although several experiments had been performed regarding α-crystallin membrane association [26,32,33,37,48], a detailed study investigating the link between the membrane’s mechanical properties and α-crystallin association at the molecular level was lacking. In this study, we investigated the modulation of the mechanical properties of the SLM with α-crystallin association using AFM—for the first time, to the best of our knowledge.

The association of α-crystallin on the lipid membrane increased with an increase in time scale. Simultaneously, α-crystallin oligomeric units were submerged inside the membrane in a process which probably is initiated by hydrophobic interaction. Note that such deep interactions are only found at the edge of the surface interaction, but they do not stand alone. A possible explanation for this might be that the surface-interacting α-crystallin aggregates depress the local bilayer, exposing the hydrophobic core of adjacent lipids, providing an opportunity for the association of α-crystallin oligomers. Once an oligomer is submerged in the SLM, it could expose the hydrophobic region of an adjacent lipid and create a binding opportunity for other α-crystallin oligomers; as a result, the α-crystallin membrane complex size would increase with incubation time. However, as reported previously, such α-crystallin submergence leads to a degree of insertion into the membrane core [59] but not membrane penetration [67]. In this study, we used the POPC membrane to prepare SLMs at room temperature (~21 °C). Since the phase transition temperature of POPC is approximately −2.5 °C [68], the SLM we investigated in this study was in the fluid phase, this being a biologically relevant phase [69]. However, earlier studies reported that the association of α-crystallin with the lens membrane is temperature-dependent [37,45]. Cobb and Petrash conjugated α-crystallin with a fluorescence tag and found that α-crystallin association with the plasma membrane increased with an increase in temperature from 25 °C to 35 °C [45]. Similarly, Mulders et al. labeled α-crystallin with [^35^S] methionine and found that α-crystallin association with the membrane increased with an increase in temperature from ~22 °C to 37 °C [37]. Based on these earlier observations [37,45], we predict that the association of α-crystallin would increase if we were to perform AFM experiments at physiological temperature (37 °C).

It has been reported that a CaCl_2_ concentration above 4 mM is capable of aggregating α-crystallin while MgCl_2_ is not capable of aggregating α-crystallin [70]. Previous studies on the α-crystallin membrane association reported that divalent cations (Ca^2+^ and Mg^2+^) do not influence α-crystallin membrane association [37]. Although our SLM preparation incorporated 5 mM CaCl_2_ in buffer B, buffer A, i.e., buffer without CaCl_2_, was used for flushing out, with the buffer being replaced before imaging. We believe that this process removed the CaCl_2_ in the solution; however, Ca^2+^ ions bound to the lipid’s headgroups (phosphate groups) [71] cannot be removed. We performed similar experiments replacing the CaCl_2_ in buffer B with 10 mM MgCl_2_ while preparing the SLM and observed a similar effect on α-crystallin membrane association (Figure 3). We believe the results presented here are not the effects of divalent cations but solely the effects of α-crystallin membrane interaction.

The α-crystallin membrane association studies reported in this paper show that SLMs prepared with CaCl_2_ and MgCl_2_ have similar topographical images and mechanical properties. Most importantly, the interaction of α-crystallin with the membrane remarkably changed the membrane’s mechanical properties. As observed by the nature of the force curves, no rupture events were seen in the deep insertion region and the close vicinity of the surface interaction region. This indicates the loss of membrane elasticity with α-crystallin association, likely making SLMs stiffer. Such an absence of rupture events in the phase-separated membranes within the maximum payload is seen in the rigid solid ordered (s_o_) phase [72] but not in the elastic liquid disordered (l_d_) region. Thus, to the best of our knowledge, our AFM results, for the first time, provide support for the idea that increased lens stiffness, as seen in aged human lenses, is due to the association of α-crystallin with the lens membrane. Nevertheless, the mechanical properties of SLMs in the region away from α-crystallin membrane association (both surface and deep) remain similar to the mechanical properties of SLMs before α-crystallin incubation. This observation suggests that α-crystallin association likely stiffens the membrane locally and plays a role in the overall stiffening of the lens that leads to presbyopia. Previously, it has been suggested that large-scale α-crystallin association with the lens membrane could lead to lens stiffening [30]; however, the mechanism is unclear. The association of α-crystallin with the membrane followed by the membrane’s loss of elastic properties provides new insights into understanding the molecular basis of lens hardening and presbyopia development. 

## Data Availability

The data will be available from the corresponding author upon reasonable request.
